# Comparison between male and female breast cancer survival using propensity score matching analysis

**DOI:** 10.1038/s41598-021-91131-4

**Published:** 2021-06-02

**Authors:** Serena Scomersi, Fabiola Giudici, Giuseppe Cacciatore, Pasquale Losurdo, Stefano Fracon, Sara Cortinovis, Rita Ceccherini, Fabrizio Zanconati, Maura Tonutti, Marina Bortul

**Affiliations:** 1 Azienda Sanitaria Universitaria Giuliano Isontina, ASUGI, SSD Breast Unit, Cattinara Hospital, Strada di Fiume 447, 34129 Trieste, Italy; 2 Unit of Biostatistics, Epidemiology and Public Health, Department of Cardiac, Thoracic, Vascular Sciences and Public Health, University of Padua, Via Loredan, 18, 35131 Padua, Italy; 3 Department of Medical, Surgical and Health Sciences, Cattinara Hospital, University of Trieste, Strada di Fiume 447, 34129 Trieste, Italy

**Keywords:** Breast cancer, Cancer therapy, Cancer, Oncology

## Abstract

Male breast cancer (MBC) is a rare disease. The few studies on MBC reported conflicting data regarding survival outcomes compared to women. This study has two objectives: to describe the characteristics of a single-cohort of MBC and to compare overall survival (OS) and disease-free survival (DFS) between men and women using the propensity score matching (PSM) analysis. We considered MBC patients (n = 40) diagnosed between January 2004 and May 2019. Clinical, pathological, oncological and follow-up data were analyzed. Univariate analysis was performed to determine the prognostic factors on OS and DFS for MBC. We selected female patients with BC (n = 2678). To minimize the effect of the imbalance of the prognostic factors between the two cohorts, the PSM method (1:3 ratio) was applied and differences in survival between the two groups were assessed. The average age of MBC patients was 73 years. The 5-year OS and DFS rates were 76.7% and 72.2% respectively. The prognostic factors that significantly influenced OS and DFS were tumor size and lymph node status. After the PSM, 5 year-OS was similar between MBC and FBC (72.9% vs 72.3%, p = 0.70) while we found a worse DFS for MBC (72.2% vs 91.4%, p  = 0.03). Our data confirmed previous reported MBC characteristics: we found a higher risk of recurrence in MBC compared to FMC but similar OS. MBC and FMC are different entities and studies are needed to understand its epidemiology and guide its management.

## Introduction

Male breast cancer (MBC) is a rare disease representing less than 1% of breast cancers, and accounting for 0.11% of all male malignancies^1–7^. Although its incidence increased over last years^3,4^, there are limited numbers of studies exclusively investigating the disease. Therefore, most available data come from observational retrospective studies limited by small sample sizes, short follow up and non-population-based design, with a consequent limitation in their interpretability. Moreover, given the lack of MBC-focused clinical studies, current recommendations on MBC management are mainly extrapolated from knowledge about female breast cancer (FBC).

Studies have reported that men with breast cancer had worse overall survival (OS) than female^6–10^: survival differences may be due to clinical differences in MBC population since MBC is usually diagnosed at older age and at a more advanced stage if compared to the female counterpart^10^. Interestingly, some studies suggested that MBC may have distinct biological characteristics compared to breast cancer in women since MBC tends to be ductal type and estrogen receptor and progesterone receptor positive^11–15^.

The purpose of the study was to investigate clinical and biological factors associated with survival outcomes of MBC patients and compare them with FBC population with the aim to assess the role of clinical characteristics in determining possible prognosis disparities. In order to reduce bias of a retrospective study, we performed one-to-three propensity score matching (PSM) analysis.

## Materials and methods

### Study population

We included patients, 18 years of age or older, diagnosed with breast cancer between January 2004 and May 2019 at the Breast Unit of University Hospital of Trieste. Data on clinical features and cancer biological characteristics and information on survival were extracted from a prospectively recorded database (DataBreast v.3, 2018). A proper broad informed consent for unspecified use of data was obtained for each patient and University Hospital approved anonymously data use according to local legislation (GDPR 679/2016, Par.26).

**Figure 1:**
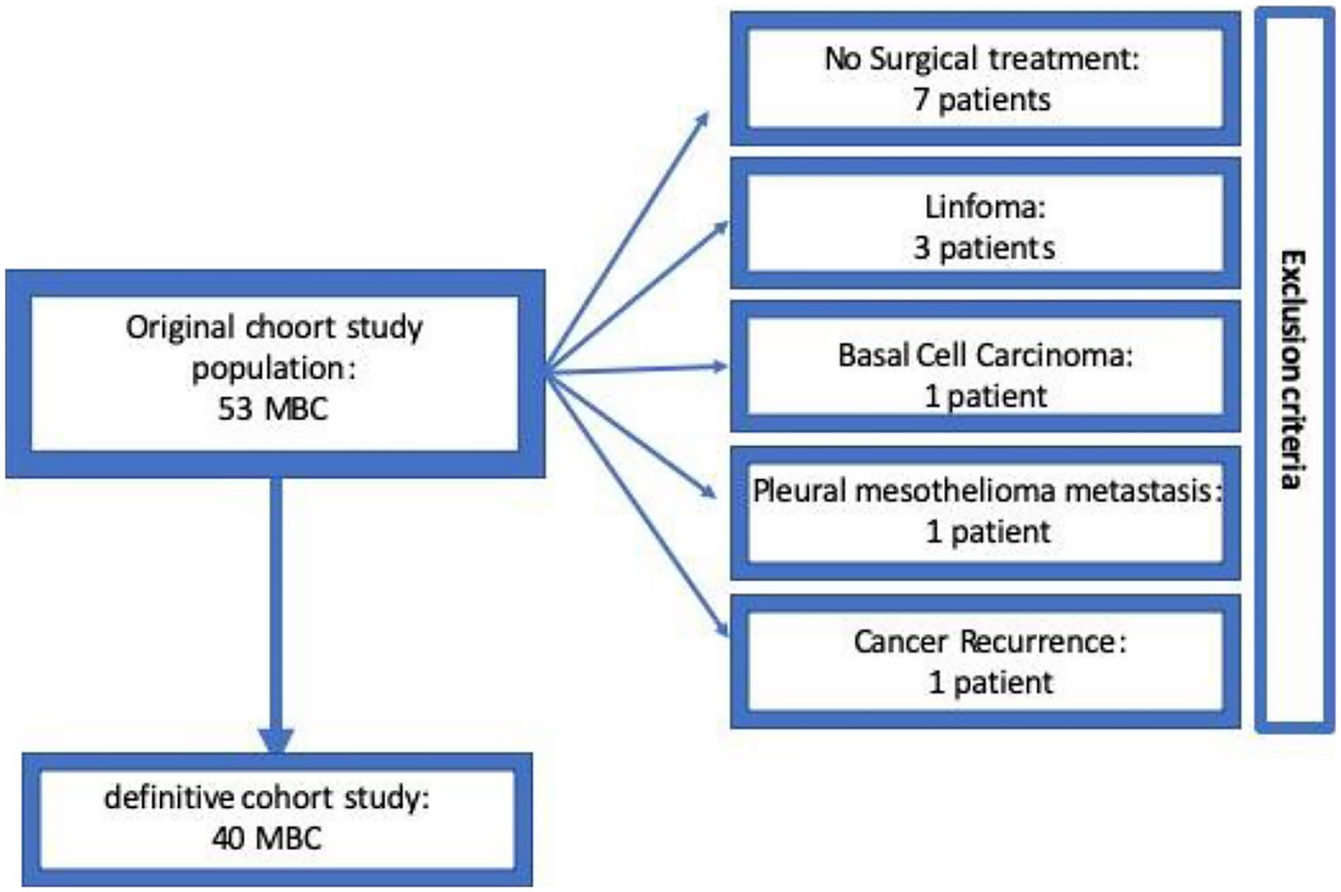
Flow-chart of inclusion and exclusion criteria.

A total of 2946 patients with breast cancer diagnosis were assessed for eligibility and 2933 fulfilled the criteria (40 MBC and 2893 FBC). Thirteen MBC patients were excluded for the following reasons (Fig. 1): 7 patients had not performed any surgical treatment; 3 patients had lymphoma diagnosis; 1 patient had basal cell carcinoma; 1 patient was metastatic; 1 patient had local recurrence from previous BC (Fig. 1).

BC diagnosis relied on mammography and axillary ultrasound followed by either fine needle aspiration or core needle biopsy. Systemic staging with PET-CT was reserved only to node positive patients and metastatic patients were excluded.

Demographic characteristics including sex, age at diagnosis, year of diagnosis, familial history of breast cancer were recorded. Clinical characteristics included tumor size, nodal status, TNM stage according to AJCC, WHO histologic type, Nottingham combined histologic grade, estrogen receptor (ER) status, progesterone receptor (PR) status, proliferative index Ki-67, human epidermal growth factor receptor 2 (HER2) status, type of surgery performed, postoperative adjuvant treatment and postoperative radiotherapy. ER, PR, and HER2 expression were based on immunohistochemistry testing. In equivocal HER2 cases, data from fluorescent in-situ hybridization assay records were used for molecular stratification. Breast cancer subtypes were grouped into the following categories according to St. Gallen 2013: luminal A cases (ER+/PR+/HER2−, Ki-67 < 20%), luminal B cases (luminal B HER2−: HER+/HER2− and at least one of Ki-67 ≥ 20% or PR−, luminal B HER2+: ER+/HER2+/any Ki-67/any PR), HER2+ (ER−/PR−/HER2+), and triple-negative breast cancers (ER−/PR−/HER2−).

### Statistical analysis

The clinical and pathological characteristics of the patients were described by means of ± standard deviation (SD) or median and range (minimum–maximum) for continuous variables, while with absolute and percentage frequencies for categorical variables. The Shapiro–Wilk test was applied to continuous variables to verify the normality of the distribution.

DFS and OS were defined respectively as the time from the date of surgery to the date of appearance of recurrence or to the date of death for each cause. Patients who did not have the event were considered to be censored at the end of the follow-up. The median follow-up was computed for censored patients, excluding patients with the events of interest (reverse Kaplan–Meyer method). The DFS and OS were estimated with the Kaplan-Meyer method and the differences in DFS and OS between the groups (case-controls) were compared using the Log-Rank test (Mantel Cox). Cox univariate regression analyses were performed for OS and DFS outcomes and hazard ratios (HR) with the relative confidence interval at 95% were reported. The assumption of proportional hazards was assessed by Schoenfeld residuals. The follow up was updated to May 2019.

Comparisons between groups (men and women) with respect to continuous variables of interest, both before and after the analysis by propensity score matching, were evaluated using the t-student parametric test or the Mann–Whitney non-parametric test according to data distribution.

The associations between the categorical variables were evaluated with the Chi-Square test or with the Fisher exact test when appropriate.

To compare MBC and FBC respect to DFS and OS, in order to control potential confounders and selection bias, we performed a sensitivity analysis using propensity score matching with the R package ‘MatchIt’ (method nearest neighbor)^16^. The patients were matched 1:3 by year of surgery, age, type of surgery, pT, pN and molecular profile.

Out of 2893 FBC surgically treated in the period, 215 were not eligible for the propensity score analysis: 83 women with neoadjuvant treatment; 114 women for whom nodal status was not available and 18 women had an incomplete molecular profile. So, 40 male patients were matched to 120 female patients.

All statistical analyses were performed using the statistical software R (the R Foundation for Statistical Computing; Version 3.5.0) and the software STATA 14.2 (StataCorp, College Station, TX). P-value values ​​less than 0.05 were considered statistically significant.

### Ethical approval

Study was conducted in accordance with the ethical standards of the institutional and national research committee and with the 1964 Helsinki declaration and its later amendments or comparable ethical standards. Data were stored anonymously in a database which is the official Eusoma database, not open access. A proper broad informed consent for unspecified use of data was obtained for each patient. Patients data were stored only anonymized, so confidentiality of the information linked to the data is guaranteed. Nobody of the researchers had access to identifying patients’ information. According to local legislation (GDPR 679/2016, Par.26), anonymized data don’t require data protection and ethical committee approvement.

## Results

Overall MBC in the present series represents 1.4% of all breast cancers. The baseline characteristics of MBC patients were reported in Table 1. Median age at diagnosis was 73.19 years old (range: 53.67–93.85). Breast Cancer Stage at diagnosis according to AJCC was stage II in 42.50% of patients. Clinical characteristics analysis showed that the vast majority of breast cancers were Ductal Infiltrating Carcinoma (90%) and hormone receptor positive (92.5%). All MBC patients were surgically treated with mastectomy. A great amount of patients (75.68%) received adjuvant endocrine therapy and 13.51% of them were treated with adjuvant chemotherapy. Median follow-up was 2.39 years (range: 0.04–11.93) years, with cancer recurrence observed in 15% of patients.

**Table 1:** Clinical and pathological characteristics of MBC patients (n = 40).

**Age (years)**	
Mean ± sd	73.52 ± 9.21
Median age (min–max)	73.19 (53.67–93.85)
**Family history of breast cancer**	
No	18 (45.00%)
First degree-relative	6 (15.50%)
Second degree-relative	3 (7.50%)
Not known	13 (32.50%)
**Type of SURGERY**	
Conservative	0 (0.00%)
Mastectomy	40 (100.00%)
**Preoperative axillary cytology**	
No	30 (75.00%)
Yes positive	9 (22.50%)
Yes negative	1 (2.50%)
**Surgery on axilla**	
Only SLNB	16 (40.00%)
Axillary dissection	22 (55.00%)
No axilla examination	2 (5.00%)
**SLNB**	
Negative	15 (65.22%)
Positive	8 (34.78%)
**N° of SLNB examination**	
1	12 (52.17%)
2	8 (34.78%)
> 3	3 (13.04%)
**N° of metastatic SLNB**	
1	6 (75.00%)
2	2 (25.00%)
> 3	0 (0.00%)
**N° of positive SLNB after AD**	
0	6 (75.00%)
1	2 (25.00%)
≥ 2	0 (0.00%)
**Tumor stage**	
in situ	3 (7.50%)
T1a-b	2 (5.00%)
T1c	16 (40.00%)
T2	14 (35.0%)
T4b	5 (12.50%)
**Nodal stage**	
N0	18 (45.00%)
N1mi	1 (2.50%)
N1a–b	16 (40.00%)
N2–N3	5 (12.50%)
**Stage TNM**	
0	3 (7.50%)
I	12 (30.00%)
II	17 (42.50%)
III	8 (20.00%)
**Grading**	
G1	6 (15.00%)
G2	22 (55.00%)
G3	11 (27.50%)
GX	1 (2.50%)
**Histotype**	
In situ	3 (7.50%)
Ductal infiltrating carcinoma	36 (90.00%)
Others	1 (2.50%)
**Ki-67**	
< 20	15 (37.50%)
≥ 20	25 (62.50%)
**Molecular profile**	
In situ	3 (7.50%)
Luminal A	15 (37.50%)
Luminal B HER2−	16 (40.00%)
Luminal B HER2+	6 (15.00%)
HER2+	0 (0.00%)
Triple negative	0 (0.00%)
**Adjuvant endocrine therapy**	
Yes	28 (75.68%)
No	4 (10.81%)
Unknown	5 (13.51%)
**Adjuvant chemotherapy**	
Yes	5 (13.51%)
No	27 (72.97%)
Unknown	5 (13.51%)
**Postoperative radiotherapy**	
Yes	6 (16.22%)
No	26 (70.27%)
Unknown	5 (13.51%)
**Cancer recurrence**	
Yes	6 (15%)
No	34 (85%)
**Death**	
Yes	12 (30%)
No	28 (70%)
**Cause of death**	
Breast cancer	5 (41.67%)
Other	7 (58.33%)
**Follow-up (in years)**	
Median (min–max)	2.39 (0.04–11.93)

*sd* standard deviation, *SLNB* sentinel lymph node biopsy, *AD* axillary dissection.

### Overall survival

**Figure 2:**
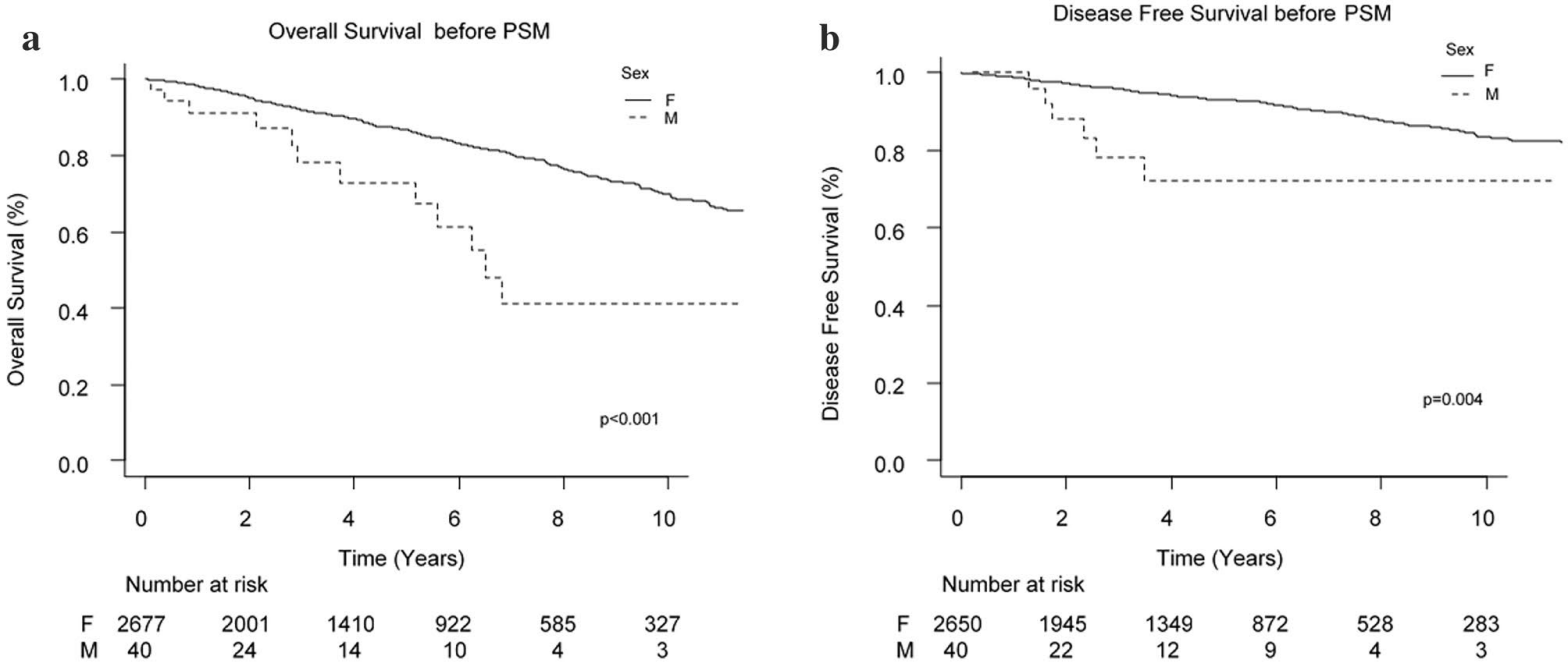
(**a**) OS before PSM. (**b**) DFS before PSM.

MBC group showed median survival of 6.5 years. Five-year survival was 76.7% (95% CI 57.20–88.10) (Fig. 2a). As shown in Table 2, stage-specific subgroup analysis demonstrated statistically significant differences in OS for patients at pT4 stage, pN+ patients and Stage III patients. In particular, stage pT4 MBC patients showed higher risk of death compared to stage pT1a-b patients (p = 0.04); patients with positive lymph nodes had higher risk of death (p = 0.05); as well as stage III MBC (0.08).

**Table 2:** Overall survival: univariate analysis.

Variabile	HR [95% CI]	p-value
**Age**	1.02 [0.95–1.09]	p = 0.57
**Tumor stage**		
T1a–b-is	Reference	
T1c	0.89 [0.09–8.99]	p = 0.92
T2	0.75 [0.08–7.18]	p = 0.80
T4	10.58 [1.05–106.67]	p = 0.04
**Nodal stage**		
N0	Reference	
N+	3.67 [0.99–13.60]	p = 0.05
**TNM stage**		
0–I	Reference	
II	2.10 [0.41–10.84]	p = 0.38
III	4.31 [0.83–22.38]	p = 0.08
**Grading**		
G1	Reference	
G2	0.81 [0.20–3.25]	p = 0.77
G3	2.29 [0.44–11.86]	p = 0.32
**Ki-67**		
< 20	Reference	
≥ 20	1.41 [0.38–5.23]	p = 0.61
**Molecular profile**		
Luminal A	Reference	
Luminal B HER-	1.06 [0.25–4.46]	p = 0.93
Luminal B HER +	2.58 [0.52–12.89]	p = 0.25

*HR* hazard ratio, *CI* confidence interval.

### Disease free survival

Disease-free survival at 5 years in MBC group was 72.2% (95% CI 47.50–86.70%) (Fig. 2b). Cancer recurrence occurred in 15% of patients (4 local–regional relapse and 2 metastases) during the first 4 years after diagnosis (median time of relapse since first diagnosis: 2.03 years). Univariate analysis was conducted and showed a strong correlation between DFS and lymph node status, being free from disease only 46.2% of MBC patients at 5 years (p < 0.001 log-rank test). MBC patients with pT2-4 tumors tended to have a worse DFS if compared to pT1 tumors (p < 0.001, log-rank test). Stage III patients showed higher recurrences with 5 out of 8 patients with progression of disease (p < 0.001 log-rank test).

### Propensity score analysis

A pool of 2678 women was considered to set up the match with the respective 40 men. We excluded from the series 83 FBC patients treated with preoperative chemotherapy, 114 FBC patients with undetermined lymph node status and 18 FBC patients with missing data about their molecular profile. The baseline clinical and pathological characteristics of the two groups are described in Table 3.

**Table 3:** Comparison of the clinical and pathological characteristics between the two groups before and after the matching procedure.

	MBC patients (n = 40)	FBC patients before PSM (n = 2678)	p-value^a^	FBC patients after PSM (n = 120)	p-value^b^
**Age (years)**					
Median (min–max)	73 (54–94)	66 (24–100)	p = 0.001	77 (46–95)	p = 0.30
**Year of surgery**					
2004–2006	9 (22.50%)	395 (14.75%)	p = 0.07	25 (20.93%)	p = 0.73
2007–2009	5 (12.5%)	397 (14.83%)		21 (17.50%)	
2010–2012	8 (20.00%)	512 (19.12%)		19 (15.83%)	
2013–2015	4 (10.00%)	538 (20.09%)		20 (16.67%)	
2016–2019	14 (35.00%)	836 (31.22%)		35 (29.17%)	
**Type of surgery**					
Conservative	0 (0.00%)	1791 (66.88%)	p < 0.001	0 (0.00%)	p = 1.00
Mastectomy	40 (100.00%)	887 (33.12%)		120 (100.00%)	
**Tumor stage**					
In situ	3 (7.50%)	272 (10.16%)	p = 0.001	6 (5.00%)	p = 0.85
T1a–b	2 (5.00%)	653 (24.38%)		10 (8.33%)	
T1c	16 (40.00%)	946 (35.32%)		41 (34.17%)	
T2	14 (35.0%)	645 (24.09%)		49 (40.83%)	
T3–T4	5 (12.50%)	79 (2.95%)		14 (11.67%)	
		83 (3.10%)			
**Nodal stage**					
N0	18 (45.00%)	1799 (67.18%)	p = 0.001	49 (40.83%)	p = 0.96
N1mi	1 (2.50%)	443 (16.54%)		50 (41.67%)	
N1a–b	16 (40.00%)	171 (6.39%)		3 (2.50%)	
N2–N3	5 (12.50%)	265 (9.90%)		18 (15.0%)	
**Grading**					
G1	6 (15.00%)	304 (11.35%)	p = 0.67	8 (6.67%)	p = 0.20
G2	22 (55.00%)	1556 (58.10%)		86 (71.67%)	
G3	11 (27.50%)	649 (24.23%)		23 (19.17%)	
GX	1 (2.50%)	169 (6.41%)		3 (2.50%)	
**Ki-67**					
< 20	15 (37.50%)	1587 (59.26%)	p = 0.006	65 (54.17%)	p = 0.07
≥ 20	25 (62.50%)	1091 (40.74%)		55 (46.83%)	
**Molecular profile**					
In situ	3 (7.50%)	276 (10.31%)	p = 0.03	6 (5.00%)	p = 0.91
Luminal A	15 (37.50%)	1121 (41.86%)		51 (42.50%)	
Luminal B HER2-	16 (40.00%)	800 (29.87%)		46 (38.33%)	
Luminal B HER2+	6 (15.00%)	155 (5.79%)		17 (14.17%)	
HER2+	0 (0.00%)	87 (3.35%)		0 (0.00%)	
Triple negative	0 (0.00%)	239 (8.92%)		0 (0.00%)	
**Cancer recurrence**					
Yes	6 (15%)	203 (7.58%)	p = 0.07	8 (6.67%)	p = 0.10
No	34 (85%)	2474 (92.42%)		112 (93.33%)	
**Death**					
Yes	12 (30%)	444 (16.59%)	p = 0.02	43 (35.83%)	p = 0.02
No	28 (70%)	2233 (83.41%)		77 (64.17%)	

*MBC* male breast cancer, *FBC* female breast cancer, *PSM* propensity score matching.

^a^p-value results before matching procedure.

^b^p-value results after matching procedure.

**Figure 3:**
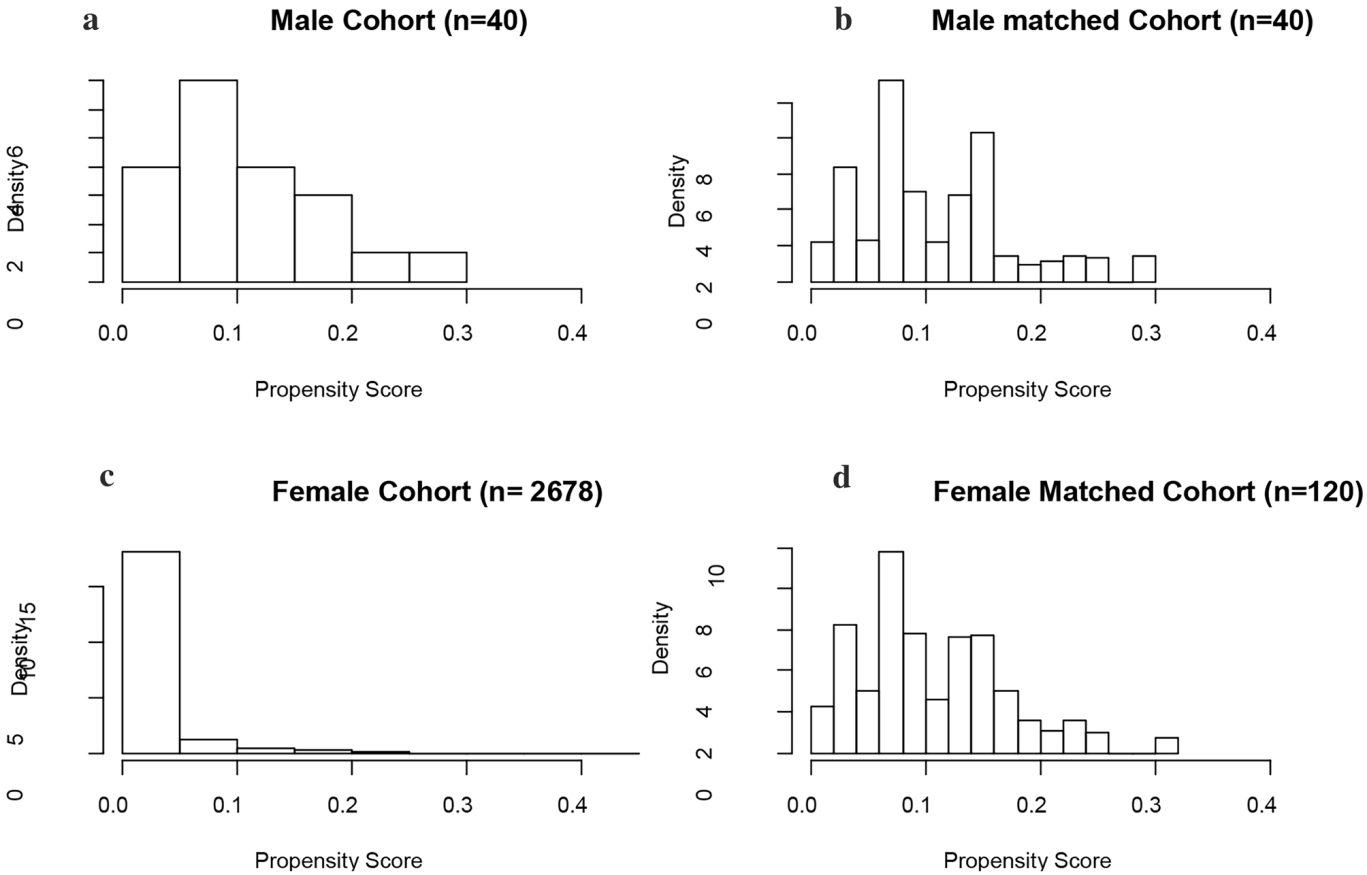
Distribution of propensity score before and after propensity score analysis. (**A**, **C**) Show the distribution of the propensity score for patients with MBC and FBC before the matching procedure, respectively. (**B**, **D**) Demonstrate the distribution of the propensity score after full propensity score matching.

Before adjustment MBC showed worse prognosis than FBC and the difference was statistically significant (p < 0.001 for OS and p = 0.004 for DFS, Log-rank test). The two groups (MBC and FBC) were substantially unbalanced by age, pT, pN, type of intervention, Ki-67 and molecular profile (Fig. 3a–c).

The patients were matched 1:3 (1 man vs 3 women) by year of surgery, age, type of surgery, pT, pN and molecular profile through propensity score analysis. The clinical and pathological characteristics of the two groups after matching are described in Table 3.

**Figure 4:**
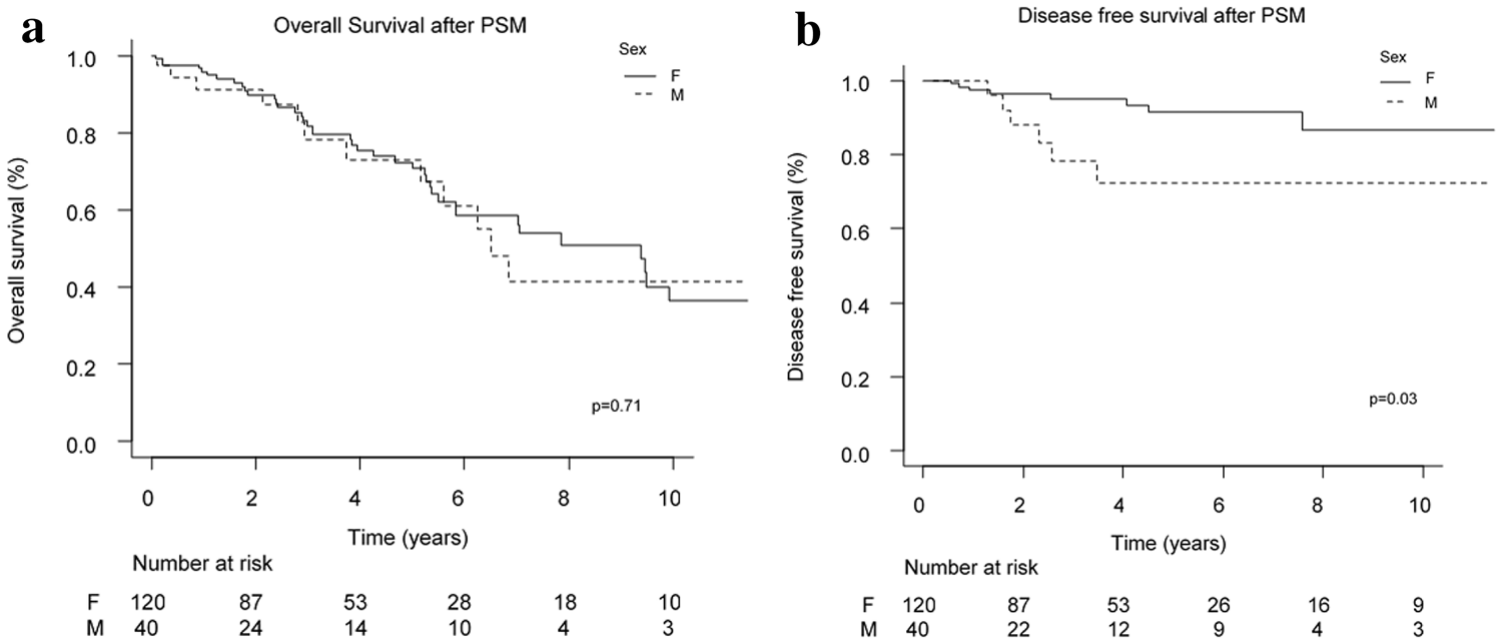
(**a**) OS after PSM. (**b**) DFS after PSM.

After matching procedure, the samples became well balanced (Fig. 3b–d) and there were no differences observed in OS between the two groups (Fig. 4a, p = 0.71, Log-rank test). In particular, 5 years after surgery, OS between male and female patients was superimposable (respectively: 0.73, 95% CI 0.50–0.87 and 0.72, 95% CI 0.62–0.81).

DFS at 5 years after surgery (Fig. 4b) for FBC was significantly higher than for MBC (respectively 5-years DFS: 0.91, 95% CI 0.82–0.96, vs 0.72, 95% CI 0.48–0.87, p = 0.03, Log-rank test).

## Discussion

The present study investigated clinical and biological factors associated with survival outcomes of MBC patients compared to FBC population. We assessed the role of clinical characteristics in determining possible prognosis disparities and investigated OS and DFS by means of propensity score matching (PSM) in order to avoid selection bias due to age, stage and clinical characteristics.

In the present single center series, MBC was confirmed to be a rare disease and represented 1.4% of all breast cancers. We observed an average age of about 73 years at diagnosis, higher than other published data and due to the demographic characteristics of the regional population^7,15,17^. Larger series have reported the median age at diagnosis of MBC to range from Mid-60 s in the US (National Cancer DataBase^18^) and 68.4 (The International Male Breast Cancer Program^15^).

At a median follow-up of 2.15 years, we observed an overall median survival of MBC patients of 6.5 years, shorter than expected from other published data, that have showed OS rates ranging from 10.4^15^ to 12.1 years^18^. These results could be easily explained if we consider that in our series median age at diagnosis was sensibly higher. El-Tamer et al.^19^ showed that age was the only significant prognostic indicator of OS and suggested that overall outcomes are related to patients age rather than the investigated disease. Moreover, being OS affected mostly by age at diagnosis, it may not be the most accurate end point of prognosis in MBC.

Histopathological features of MBC found in the current cohort are similar to those presented in other series^13,20,21^: we observed mostly infiltrating ductal carcinoma (90%) with ER and PR receptor positivity in 100% of cases. Yadav et al.^18^, out from a series of 10.873 patients derived from the National Cancer DataBase, showed a rate of ER positivity of approximately 89%, and Wang et al.^17^ registered ER positivity rates of 83.9% on 1.816.733 patients out from the same series. Experience derived from The International Male BC Program^15^, that relies on centrally reviewed clinical data and tumor samples, showed ER positivity rates of 99.3%, comparable to our findings. As previously reported^18^, ER positivity tends to be higher in studies that applied uniform methods of receptor status assessment and classification across their study participants, so, not surprisingly, our single center data showed higher rate of ER positivity, consistent with data coming from a series of centrally reviewed specimens^15^. Given the high rate of ER positivity in MBC across retrospective studies, Reinisch et al.^22^ designed a phase 2 randomized trial to compare changes in estradiol levels and quality of life in MBC patients treated with adjuvant endrocrine therapy.

We found that the great majority of MBC patients showed a higher stage of disease at diagnosis with 47.5% of cases presented at pT2-4 stage and 55% with positive axillary lymph nodes. At univariate analyses it was showed that prognosis worsened significantly in stage pT4 patients HR: 10.58 (95% CI 1.05–106.67; p = 0.04); in pN+ patients HR: 3.67 (95% CI 0.99–13.60; p = 0.05); and in stage III patients HR 4.31 (95% CI 0.83–22.38; p = 0.08). These data are consistent to other published^7,10,15,23^.

In contrast with the two largest studies^18,24^, we found that a higher grade of tumor was not associated with poor OS. Similar results were showed by Cardoso^15^ and a small Swedish study^25^.

Analyzing survival disparities in male and female breast cancer population, before PSM analysis we observed a worse prognosis in terms of reduced OS in MBC patients than in FBC ones (p < 0.001) and similar results were showed for DFS (p = 0.004).

In order to determine if the baseline inhomogeneity of the two cohorts of patients was the only feature influencing survival and in order to investigate if sex was an independent factor in determining survival differences, we performed propensity score matching analysis.

After matching the two cohorts were homogeneous and balanced for most of the clinical pathological features considered and we found no significant differences in terms of OS between the two groups (p = 0.71). We only found a statistically significant difference (p = 0.03) with regard to DFS. 5 years after surgery the DFS in women was greater than that of men, respectively: 0.91, 95% CI 0.82–0.96, vs 0.72, 95% CI 0.48–0.87.

These data are consistent with other published. Miao et al.^7^ showed, in a series derived from 6 population-based cancer registries, that men had worse OS than women but, after adjusting for region, age and year of diagnosis and stage they observed no differences in 5 years excess mortality of men versus women. So, they concluded that male sex is not an independent risk factor of poor outcome after breast cancer. Other series^19^ showed that sex was not significant on OS and that men showed even a significant better disease specific survival. On the other hand, a very large series published in 2019^17^ from analysis of National Cancer DataBase showed that disparity in mortality remained even after adjustment for age, race, clinical and treatment issues.

At our knowledge, other published studies^26,27^ carried out propensity score matching to adjust potential confounding factors. In the series of Wang et al.^28^ their balanced group had a ratio of approximately two FBC to every MBC, and showed a Ten Years OS of 58.3%, with FBC with superior OS than MBC even after matching, in contrast with our findings.

The current study is limited by the small number of patients, a short median follow-up (only 2.39 years) and its reliance on a retrospective database, but being a single center series with homogenous pathological assessment with application of propensity score matching with 3:1 pairing give strength to the analysis.

## Conclusions

The study found that MBC patients had worst prognosis than FBC population. After propensity score analysis, we found no differences in terms of OS in MBC patient than their female counterpart and we registered that DFS in women was higher than that of men. Future prospective randomized studies on large cohorts of patients are needed, and inclusion of male patients in breast cancer clinical trials must be encouraged in order to obtain information to guide treatment decision in this population of patients.
